# Economic statistical model of the np chart for monitoring defectives

**DOI:** 10.1038/s41598-023-40151-3

**Published:** 2023-08-14

**Authors:** Salah Haridy, Batool Alamassi, Ahmed Maged, Mohammad Shamsuzzaman, Ali Al Owad, Hamdi Bashir

**Affiliations:** 1 Department of Industrial Engineering and Engineering Management, College of Engineering, University of Sharjah, Sharjah, UAE; 2 Benha Faculty of Engineering, Benha University, Benha, Egypt; 3 Department of Advanced Design and Systems Engineering, City University of Hong Kong, Kowloon, Hong Kong; 4 Department of Industrial Engineering, Faculty of Engineering, Jazan University, Jazan, Saudi Arabia

**Keywords:** Statistics, Mechanical engineering

## Abstract

When monitoring manufacturing processes, managing an attribute quality characteristic is easier and faster than a variable quality characteristic. Yet, the economic-statistical design of attribute control charts has attracted much less attention than variable control charts in the literature. This study develops an algorithm for optimizing the economic-statistical performance of the np chart for monitoring defectives, based on Duncan’s economic model. This algorithm has the merit of the economic model to minimize expected total cost, and the benefit of the statistical design to enhance the effectiveness of detecting increasing shifts in defectives. The effectiveness of the developed np chart is investigated under different operational scenarios. The results affirm a considerable superiority of the proposed np chart over the traditional np chart. Real-life data are used to demonstrate the applicability of the proposed np scheme, in comparison to the traditional np chart.

## Introduction

Control charts are widely employed to establish and monitor statistical control of a process. It is an effective tool for assessing process parameters. Control charts design requires the selection of a sample size (*n*), a sampling interval (*h*), and the control limits (upper control limit (*UCL*) and lower control limit (*LCL*)). The process of selecting the control chart parameters is generally known as the control chart design^1^. Various analysis techniques are required to model shifting processes^2–5^. Different design models of control charts have been developed in the literature. They are usually grouped into three main classifications: statistical, economic, and economic-statistical designs. Conventionally, the control limits of the statistical charts are calculated to be equal to $\mu \pm 3\sigma$, where $\mu$ and $\sigma$ are the mean and standard deviation of the process. The sample size (*n*) is usually suggested to be equivalent to 4 or 5 and the sampling interval (*h*) is selected based on production rate and/or availability of inspection resources^6,7^. In practice, these heuristically designed Shewhart charts are the most common since they are simple to understand and use with minimal operator training needed. Even though Shewhart charts assess the expenses implicitly by selecting *n* and *h*, the resultant charts are not guaranteed to be economically optimal.

The performance of statistically designed charts is typically determined in terms of the Average Run Length (*ARL*) or Average Time to Signal (*ATS*) where *ATS* = *ARL* × *h*^8,9^. The out-of-control *ARL* can be defined as the average number of samples required to detect a process shift after it occurs^10–12^. Similarly, the out-of-control *ATS* can be defined as the average time required to detect a process shift after it occurs^13–15^. The establishment of a cost-related design of control charts is needed due to the lack of any measure that directly represents costs. Therefore, the economic design of control charts was introduced to evaluate the performance in terms of monetary value. The chart parameters, *n, h*, *LCL* and *UCL*, are usually searched to maximize the total expected profit or to minimize the total expected cost^16–18^.

Both statistical and economic designs have their own strengths and weaknesses. Statistical designs of the control charts result in high detection power and low Type I error but then the related expenses are not investigated. On the other hand, the primary focus of the economic designs is on minimizing the cost associated with the poor quality. While economic design aims to achieve cost efficiency, it ignores the control of false alarm rate which in turn can deteriorate the detection effectiveness of the control chart^19–22^. Thus, the initiation of a model that combines the advantages of both designs was really required. The economic-statistical design was initially introduced by Saniga^20^. It successfully reduced the disadvantages of both economic and statistical designs and resulted in minimizing the expected cost, while improving the detection effectiveness of the control charts. The purpose of economic-statistical design of control charts is to minimize the expected total cost under statistical constraints^23^. Economic-statistical designs are generally more expensive than economic designs because of the extra statistical constraints. However, such statistical constraints successfully reduce the process variability and enhance product quality. Economic-statistical design is used to avoid the disadvantages of economic and statistical designs, by utilizing an economic objective function which is usually a function of cost parameters and out-of-control *ATS*, in addition to constraints on the false alarm rate (${ATS}_{0}$) and/or inspection rate (*r*)^24–27^.

Since the introduction of the economic design of control charts, several economic models have been proposed in the literature^28^. In Duncan’s model, an economic design of $\overline{X }$ chart was established to determine its optimal parameters *n*, *h*, *LCL*, and *UCL*. The parameters were identified to minimize the expected total cost per operational cycle. Recently, many authors considered the economic-statistical design of control charts. Safe et al.^29^ designed a multi objective genetic algorithm for economic-statistical design of $\overline{X }$ control chart. The findings of the study provide a listing of feasible optimal results and graphical interpretations that proves the flexibility and adaptability of the proposed approach. Naderi et al.^30^ presented an economic-statistical design of $\overline{X }$ chart under Weibull model using correlated samples under non-uniform and uniform sampling. The objective of the study was to optimize the average cost while considering different combination of Weibull distribution parameters estimates. Similarly, Lee and Khoo^31^ investigated the economic-statistical design of double sampling (DS) *S* chart. The optimum DS *S* chart design parameters were found by minimizing the cost while keeping statistical constraints in consideration. The study shows that the DS *S* chart is better than Shewhart-type chart, with a minimal cost increase and higher statistical performance. Further, Katebi and Pourtaheri^32^ proposed an economic-statistical design of exponentially weighted moving average (EWMA) control chart to monitor the average number of defects. Evaluation of the economic performance in terms of the hourly expected cost and the statistical performance in terms of *ARL* was conducted using the cost function developed by Woodall^33^. Using the design of experiment (DOE), a sensitivity analysis was undertaken to explore the effect of the factors on the result of the economic-statistical model. The results revealed that EWMA outperforms the typical Shewhart control chart. The DOE results showed that the most effective parameters are the size of the shift and assignable cause rate of occurrence. In addition, Saleh et al.^34^ assessed the in-control performance of EWMA in terms of standard deviation of *ARL*. Lu and Reyonlds^35^ created an Exponentially Weighted Moving Average (EWMA) control chart to monitor the mean of autocorrelated processes because process autocorrelation has a significant impact on control chart performance. Namin et al.^36^ developed an economic-statistical model for the acceptance control chart (ACC) where there is uncertainty in the parameters of some processes. Tavakoli and Pourtaheri^37^ proposed an economic-statistical design of the variable sample size (VSS) multivariate Bayesian chart. The developed VSS multivariate Bayesian chart was found to perform better than the T^2^ multivariate Bayesian chart. Although there has been a lot of interest in the economic-statistical design of control charts in the literature, there have only been a few articles that deal with the economic-statistical design of control charts^36,38,39^, mostly on the design of *p* control chart^40^. In general, the economic-statistical design of the attribute control charts has been extremely oversighted in the literature compared with variable charts, although in practice they have wider applications. The attribute chart popularity comes from the ease of understanding and implementation, compared with variable charts^41^.

The motivation behind this study stems from the observation that while variable control charts received significant attention in the literature, attribute charts have been relatively overlooked in terms of their economic-statistical design despite their widespread applications and ease of implementation. The economic-statistical design of control charts aims to optimize the chart’s parameters to minimize the expected total cost, while simultaneously enhancing its statistical properties and detection effectiveness for detecting out-of-control scenarios. Traditionally, control charts have been designed using statistical considerations, such as setting control limits based on specific Type I error probability. However, these charts do not explicitly address the economic aspects of quality control. On the other hand, economic designs of control charts focus primarily on minimizing costs associated with poor quality but may neglect the control of false alarm rates, which can deteriorate the detection effectiveness.

To bridge this gap, this research proposes an algorithm for optimizing the economic-statistical design of the np chart for monitoring shifts in fraction nonconforming (*p*) based on Duncan’s model, which is a well-established model in the literature and due to its elegance in terms of considering all essential costs needed in manufacturing applications. In this design, two statistical constraints on the false alarm rate and the inspection rate are incorporated to overcome the weakness of the economic design. By optimizing the design of the np chart, the researchers aim to improve the overall efficiency of quality control processes in industries. The developed optimal np chart is compared with its traditional np counterpart, EWMA chart and synthetic chart in a real-world application within the water bottle manufacturing industry. In addition, a sensitivity analysis is performed to investigate the impact of the design specifications and process shift distributions on the performance of the proposed np chart under different operational scenarios.

The rest of the article is organized as follows: “Methodology” section explains the proposed methodology, including the operation and optimization of the chart. In “Comparative studies and sensitivity analyses” section, comparisons and sensitivity analyses are carried out. Finally, “Conclusions” section concludes the article by summarizing the findings, discussing the theoretical contributions, exploring practical implications, and providing suggestions for future research directions.

## Methodology

The operation and optimization design of the proposed np control chart is explained in this section.

### Operation of the np chart

The np chart is a type of attribute chart that is employed to monitor the number of defectives (*d*) in a sample. When using an np chart to monitor a process, the process is assumed to be in control if *LCL* ≤ *d* ≤ *UCL*, where *LCL* and *UCL* are the np chart’s lower and upper control limits, respectively. That is, if *d* < *LCL* and/or *d* > *UCL*, the control chart will declare an out-of-control condition. However, the focus of this research is to develop an np chart that detects the increasing shift in defectives since a decreasing shift of defectives, when *d* < *LCL*, is actually a desired goal and not of any risk. Consequently, the *LCL* is not considered as a decision variable in this research.

If the in-control fraction nonconforming *p*_0_ of a process shifted to an out-of-control fraction nonconforming *p*, then the relationship is defined as follows:

1 $$p=\delta \times {p}_{0,}$$

where δ > 1 means that the process is out-of-control and *δ* = 1 indicates that the process is in control (i.e., *p* = *p*_0_). The monitoring process using np chart can be described as follows:

1. A sample of *n* units is obtained at the end of each *h*, and *d* is counted for that sample.
2. On the np chart, the counted *d* is plotted for each sample.
3. If *d* > *UCL*, then an out-of-control signal will be produced. Otherwise, the process considers in-control, and return to step 1.

The following assumptions are taken into consideration in this research. First, the procedure begins with an in-control condition where the number of defectives follows a binomial distribution. Second, the random shift follows a uniform and Rayleigh distributions. Third, in-control fraction nonconforming (${p}_{0}$) is known. Fourth, upward shifts in *p* are only considered. Finally, it is assumed that the out-of-control condition is occurred due to a single assignable cause and the occurrence of the single assignable cause follows a homogeneous Poisson distribution with mean *ε*^7^.

As mentioned earlier, the economic-statistical model in this research is developed based on Duncan’s model, which considers two main design specifications: process specifications and cost specifications. These design specifications are shown in Table 1.

**Table 1:** Design specifications of the economic statistical model.

Process specifications	${p}_{0}$	In-control fraction nonconforming. It is calculated from the historical data examined during in-control process
	*τ*	Minimum allowable in-control ${ATS}_{0}$. It is selected depending on the false alarm rate specifications
	*r*	Inspection rate. It is estimated based on the accessibility of resources like workforce and equipment
	${\delta }_{max}$	Maximum shift in fraction nonconforming. It is decided based on the maximum shift that the user wishes to detect
	*ε*	Assignable cause rate of occurrence
	*e*	Time to estimate observed data of a sample. It can be easily estimated from a field test
	*D*	Time between detecting the out-of-control condition and removing the assignable cause
Cost specifications	*b*	Sampling fixed cost
	*c*	Sampling variable cost
	*W*	Cost of locating and resolving an assignable cause
	*T*	False alarm examining cost
	*M*	Cost incurred due to the increasing *p* shift

### Optimization design of the np chart

The suggested optimization algorithm for the np chart is presented in this section. The design algorithm optimizes the decision variables, *n, h,* and *UCL* in order to minimize *ETC*. The economic-statistical design of the np control chart proposed in this study can be formulated as follows:

$$\begin{array}{*{20}c} {{\text{Objective}}\;{\text{function}}:} & {{\text{Minimize}}\;ETC} \\ \end{array} ,$$

2 $$\begin{array}{*{20}c} {{\text{Constraints}}:} & {ATS_{0} \ge \tau ,} \\ \end{array}$$

3 $$\mathrm{r }=\frac{n}{h},$$

$$\begin{array}{*{20}c} {{\text{Independent decision variables}}:} & n \\ \end{array} ,$$

$$\begin{array}{*{20}c} {{\text{Dependent decision variables}}:} & {h\;{\text{and}}\;UCL} \\ \end{array} .$$

In this optimization model, *n* is an independent variable, while *h* and *UCL* are dependent variables on values of *r* and *τ,* respectively. The parameter, *r* is the inspection rate, and it is equal to the sample size divided by the sampling interval. The constraint on *r* in Eq. (3) ensures that the resources are fully utilized to improve the detection effectiveness of the control chart. The measure *ATS*_0_ in Eq. (2) is the in-control average time to signal. It is equal to *h*/*α*, where *α* is Type I error probability that the np chart will show an out-of-control signal even though the process is actually in-control as shown in Eq. (4). On the other hand, *ATS* is the out-of-control average time to signal and equivalent to *h*/(1 − *β*) where *β* is the Type II error probability that the np chart states that the process is in-control when it is actually out-of-control as illustrated in Eq. (5). The values of *α* and *β* can be determined as follows:

4 $$\alpha =\mathrm{Pr}\left\{d>UCL\right\}=1-\sum \limits_{i=0}^{UCL}{C}_{i}^{n}(1-{p}_{0}{)}^{n-i}{{p}_{0}}^{i},$$

5 $$\beta =\mathrm{Pr}\left\{d\le UCL\right\}=\sum \limits_{i=0}^{UCL}{C}_{i}^{n}(1-p{)}^{n-i}{p}^{i}.$$

In the optimization design, *ATS*_0_ is considered as a constraint while *ATS* is incorporated into the objective function *ETC* which needs to be minimized. This guarantees that the optimal np chart will have a smaller *ATS* across the entire range of shifts, and eventually leads to better detection effectiveness. The objective function *ETC* is computed as follows:

6 $$ETC=\underset{1}{\overset{\delta max}{\int }}\delta \cdot L\left(\delta \right)\cdot {f}_{\delta }\left(\delta \right)d\delta ,$$

where ${f}_{\updelta }\left(\updelta \right)$ is the probability density function of the shift. The cost per unit time, $L\left(\delta \right)$ for a given *p* shift (in Eq. 6) is estimated based on Duncan’s^28^ economic model as follows:

7 $$L\left(\delta \right)=\frac{b+cn}{h}+\frac{\left[\frac{h}{1-\beta }-\frac{h}{2}+\frac{\varepsilon {h}^{2}}{12}+en+D\right]M\varepsilon +\frac{\alpha T}{h}+\varepsilon W}{1+\varepsilon [\frac{h}{1-\beta }-\frac{h}{2}+\frac{\varepsilon {h}^{2}}{12}+en+D]}.$$

**Figure 1:**
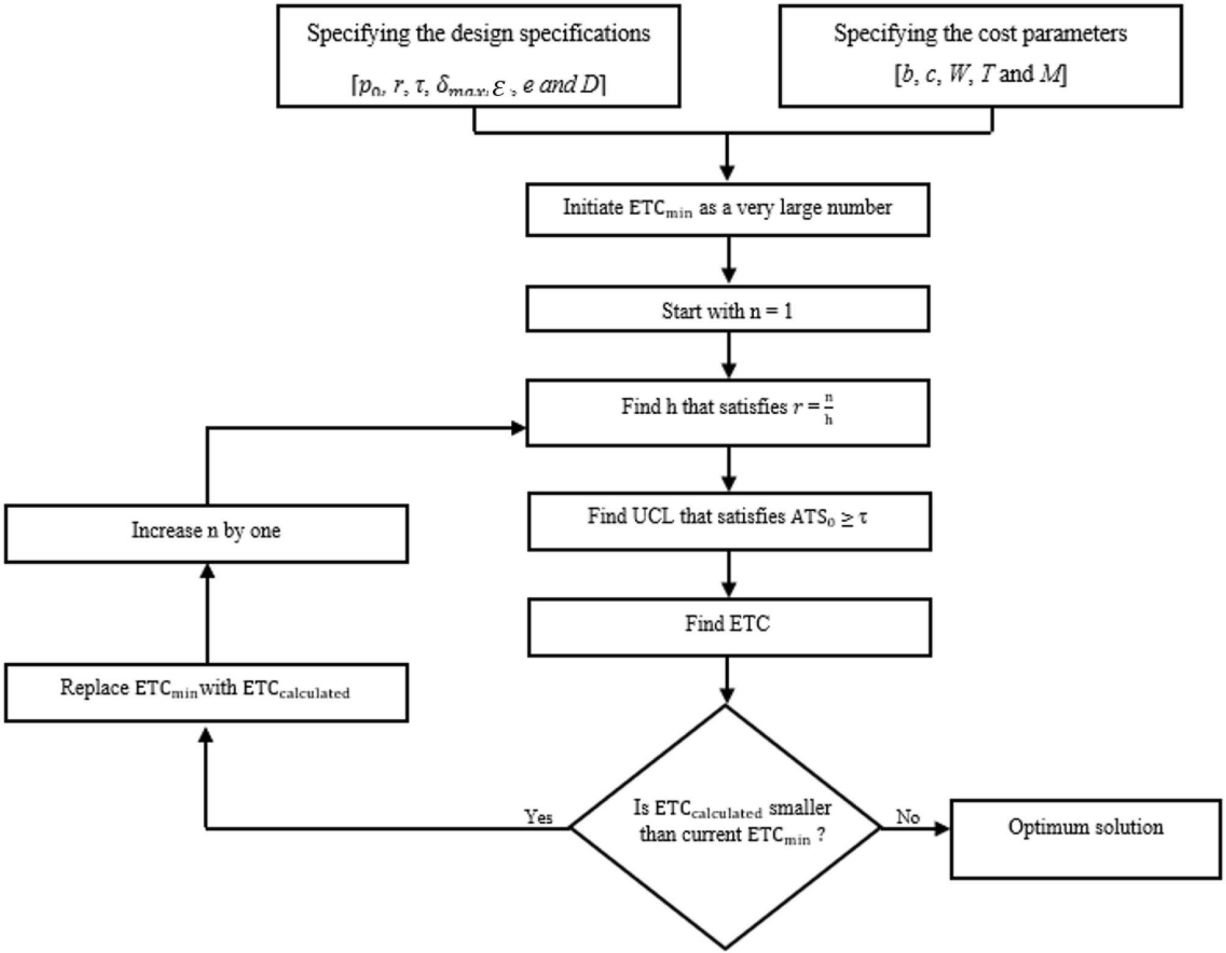
Optimization algorithm flowchart.

The optimization model identifies the optimal values of *n*, *h* and *UCL* that minimize *ETC* throughout a shift domain of ($1$≤ *δ* ≤ *δ*_*max*_) while ensuring that ${ATS}_{0}$ is no less than a specified value of* τ.* Figure 1 explains the optimization process of the np chart. It can be explained as follows:

1. Start by specifying the design specifications and the cost parameters.
2. Initiate the variable ${ETC}_{min}$ as a very large number (${ETC}_{min}$ will be used to keep track of the *ETC’s* minimum value).
3. Begin by raising *n* by one at a time, beginning with *n* = 1.
4. Find *h* that satisfies constraint (3) for each *n*.
5. For every set of (*n*, *h*), search the value of *UCL* that meets constraint (2).
6. Find the *ETC* value that corresponds to the related *n*, *h*, and *UCL*.
7. Replace *ETC*_*min*_ by the calculated *ETC* if the calculated *ETC* is smaller and save the current *n*, *h*, and *UCL* values as a temporary optimal solution.
8. Step 4 is repeated for each *n* value trial until *ETC* cannot be decreased anymore. The optimal np decision variables will generate the lowest *ETC* while satisfying requirements (2) and (3).

It is worth mentioning that the traditional np chart in this study refers to an np chart designed based on the proposed economic-statistical model and utilizing the same optimization algorithm. However, that the traditional np chart only adjusts the *UCL* to maintain *ATS*_0_ greater than *τ*, without optimizing *n* and *h*. Instead, these two parameters (*n* and *h*) are predetermined by the user. In contrast, the optimal np chart is designed to optimize all charting parameters (*UCL*, *n*, and *h*) through the above-mentioned optimization procedure.

The proposed economic-statistical optimization algorithm results in the best optimal combination of the charting parameters for the np chart. The design algorithm is coded using C programming language. In a couple of seconds, a personal computer can complete the optimization design of the np chart.

### EWMA and synthetic control charts

The Exponentially Weighted Moving Average (EWMA) and synthetic control charts exhibit high sensitivity to small and moderate shifts in the fraction nonconforming (*p*) when compared to the np chart^7,9,42^. The EWMA chart differs from the np chart as it takes into account the real-time accumulation of all samples, rather than solely relying on the latest sample^43,44^. The plotting statistic *E*_*t*_ is updated for each *t*th sample as follows^45^:

8 $${E}_{t}=\lambda \left({d}_{t}-{d}_{0}\right)+\left(1-\lambda \right){E}_{t-1},$$

where ${d}_{t}$ is the number of nonconforming items in the *t*th sample, *d*_0_ (= *n* × *p*_0_) is the in-control fraction nonconforming, and $\lambda$ ($0 < \lambda \le 1$) is the smoothing parameter. Typically, *E*_0_ and λ are set as zero and 0.1, respectively^7^. If the value of *E*_*t*_ exceeds the control limit *H* of the EWMA control chart, the process is considered out of control.

On the other hand, the synthetic chart combines the functionalities of the conforming run length (CRL) and np charts^46,47^. Consequently, it utilizes two charting parameters: the warning limit *w* (for the np sub-chart) and the lower control limit *L* (for the CRL sub-chart). The *CRL* represents the number of samples between two successive nonconforming samples, including the nonconforming sample at the end^9,48^. If *d*_*t*_ ≤ *w* in a *t*th sample, the process is declared as in control. However, if *d*_*t*_ > *w*, the sample is deemed nonconforming, and the value of *CRL* needs to be determined and compared with *L*. If *CRL* ≥ *L*, the process is in-control. Otherwise (i.e., if *CRL* < *L*), the process is considered as out-of-control.

In this study, the *n* and* h* of both the EWMA and synthetic charts satisfy constraint (3). The *H* of the EWMA chart is adjusted to fulfil constraint (2) and produce the smallest *ETC*. The value of *λ* is set as 0.1 as considered by Montgomery^7^. Similarly, the design of a synthetic chart is to find the best combination of the *w* and *L* so that the chart gives the minimum *ETC* and meanwhile meets constraint (2).

## Comparative studies and sensitivity analyses

This section compares the overall performance of the optimal np control chart with that of its competitors in terms of *ETC*, while the detection speed at each shift point is evaluated using the out-of-control *ATS*.

### Comparative study using real application

**Figure 2:**
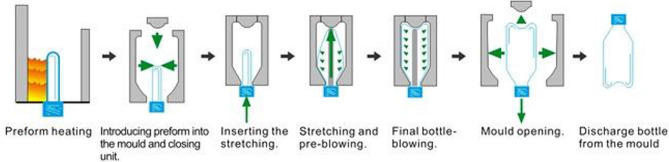
Bottle manufacturing process (Plastic Technologies, 2021).

This section demonstrates the application and design of the optimal np chart using a real-life data in water bottle manufacturing. Quality control is a process that ensures that the specifications of product or service are met. Majority of statisticians approved that the design of control charts should be implemented in two stages: Phase I, where the main goal is to collect sufficient data to evaluate the process stability, and Phase II, where monitoring and controlling the actual process is performed. In Phase I, the data are employed to design a Shewhart np chart for estimating the in-control process parameters. The manufacturing of water bottles starts from the blow moulding where the Polyethylene Terephthalate (PET) is heated and then placed in a long thin tube mould. It is then transferred to bottle shaped mould where a thin steel rod fills the tube with highly pressured air to shape the bottle. Subsequently, the mould must be cooled quite fast, so the newly shaped component is appropriately set. Once the bottle has cooled and set, it is ready to be removed from the mould. Finally, the bottle is filled, capped, and labelled. Figure 2 shows the different stages of bottle manufacturing.

**Figure 3:**
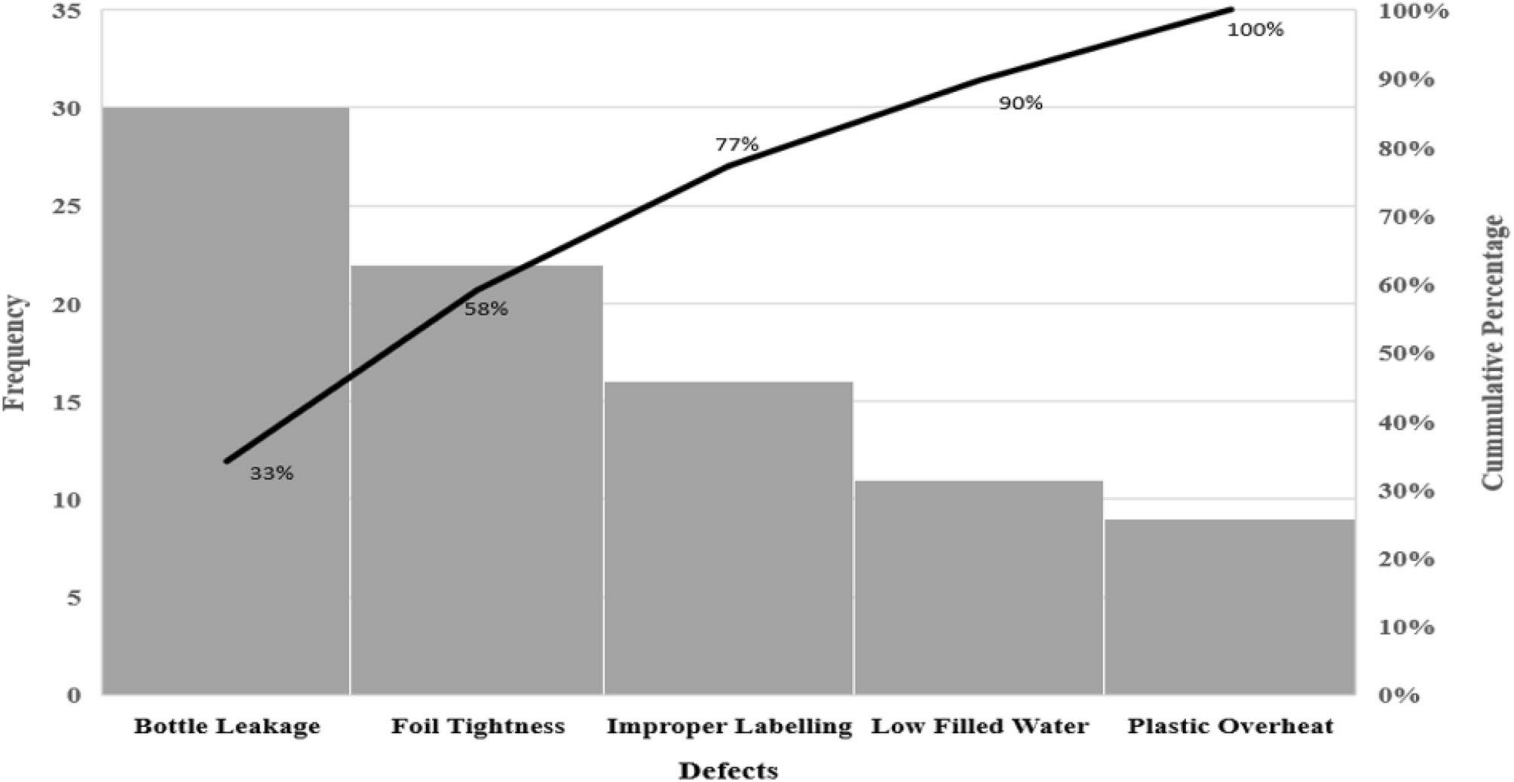
Pareto chart for water bottle defects.

A water bottle can be classified as defective if it exhibits any of the following issues: foil tightness, uncentered labeling, plastic overheating, leakage, or low water filling level. To identify the defects with the highest frequency leading to defective products, we constructed a Pareto chart. Figure 3 illustrates that approximately 77% of defective units result from the first three defects: bottle leakage, foil tightness, and improper labeling. Therefore, a bottle is deemed defective if any of these three defects are present. The decision to focus on the three defects with the highest frequency was made by the quality control engineer (QC) at the water bottle manufacturing company. By targeting the most frequently occurring defects, the company can allocate resources and efforts more effectively, address the root causes of the most impactful issues, and achieve significant improvements in overall product quality. It is worth mentioning that if the QC engineer decided to consider all five defects, the estimated in-control fraction nonconforming (*p*_0_) would change. However, the optimization algorithm proposed in this paper can still be applied straightforwardly to design the optimal np control chart, ensuring effective monitoring of the number of defective bottles.

**Figure 4:**
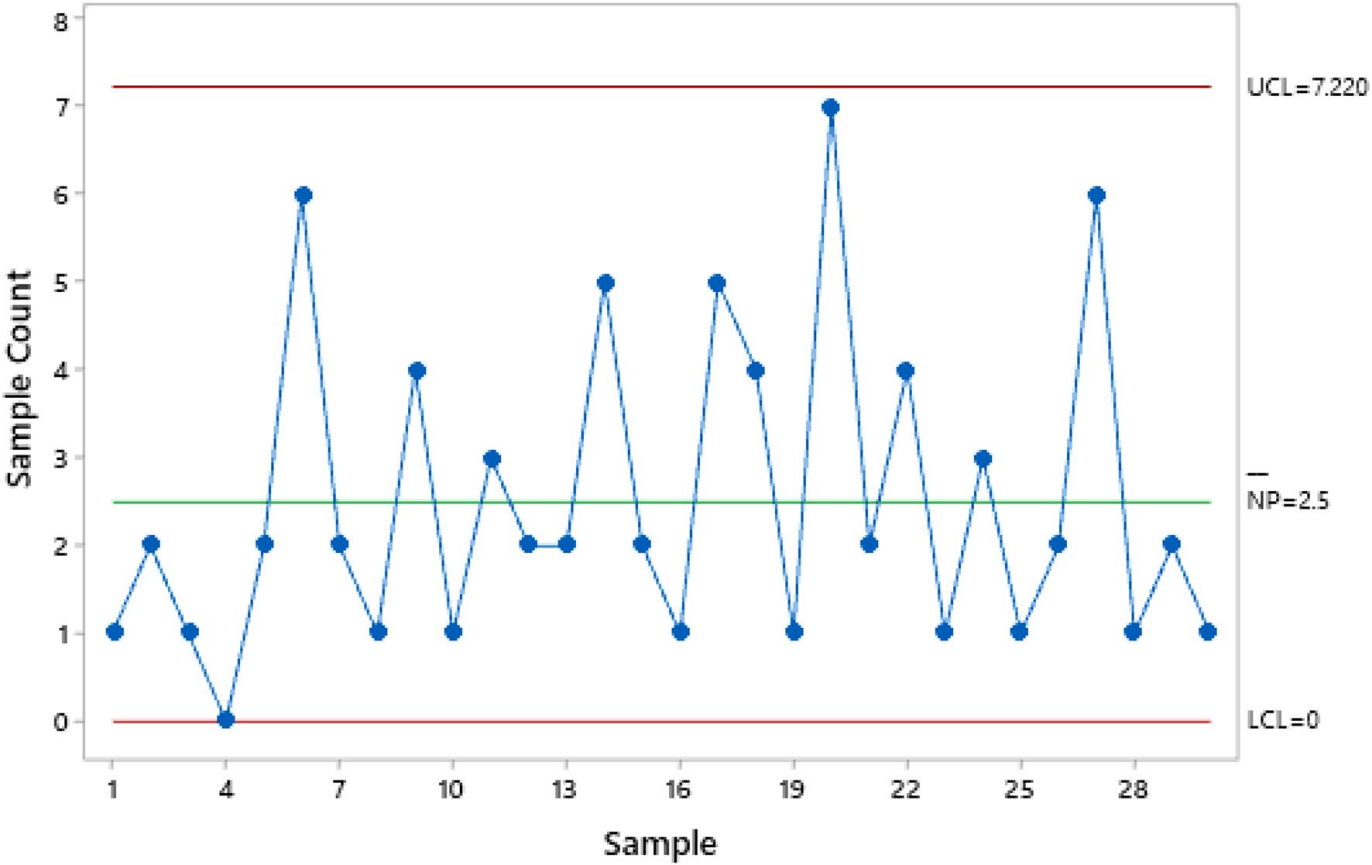
Shewhart np chart in Phase I.

The aim is to detect a rise in the fraction nonconforming *p* of the water bottles production. 30 samples (*m* = 30) with a sample size *n* of 250 were collected in Phase I. This phase aims at ensuring that the process is statistically in-control and estimating ${p}_{0}$. A Shewhart np chart is designed as shown in Fig. 4, which shows that the 30 sample points are all plotted within the control limits (*UCL* = 7.220, *CL* = 2.5, and *LCL* = 0). Since the process is statistically in-control, the fraction nonconforming ${p}_{0}$ can be estimated as follows:

8 $${p}_{0}=\frac{{\sum }_{i=1}^{m}{d}_{i}}{m\times n}=\frac{75}{30\times 250}= 0.01.$$

Most of the design and cost specifications were decided in consultation with the quality engineer. A list of the values of all specifications needed to design the np charts are shown below.

- *p*_0_ = 0.01.
- *τ* = 300 days.
- *r* = 50 units per day.
- $${\delta }_{max}$$ = 10.
- $$\varepsilon$$ = 0.05 occurrences per day.
- *e* = 0.0125 day.
- *D* = 1 day.
- *b* = 1 $.
- *c* = 0.01 $.
- *W* = 300 $.
- *T* = 100 $.
- *M* = 10,000 $.

The traditional, optimal np, EWMA and synthetic control charts are designed in Phase II. The design parameters obtained for these four charts, along with their respective performance in terms of *ETC*, are as follows:

Traditional np chart: *n* = 50, *h* = 1, *UCL* = 3 and *ETC* = 103.88.
Optimal np chart: *n* = 65, *h* = 1.3, *UCL* = 3 and *ETC* = 89.06.
EWMA chart: *n* = 50, *h* = 1, *H* = 1.210, *λ* = 0.2 and *ETC* = 81.95.
Synthetic chart: *n* = 50, *h* = 1, *w* = 2, *L* = 20 and *ETC* = 103.05.

**Figure 5:**
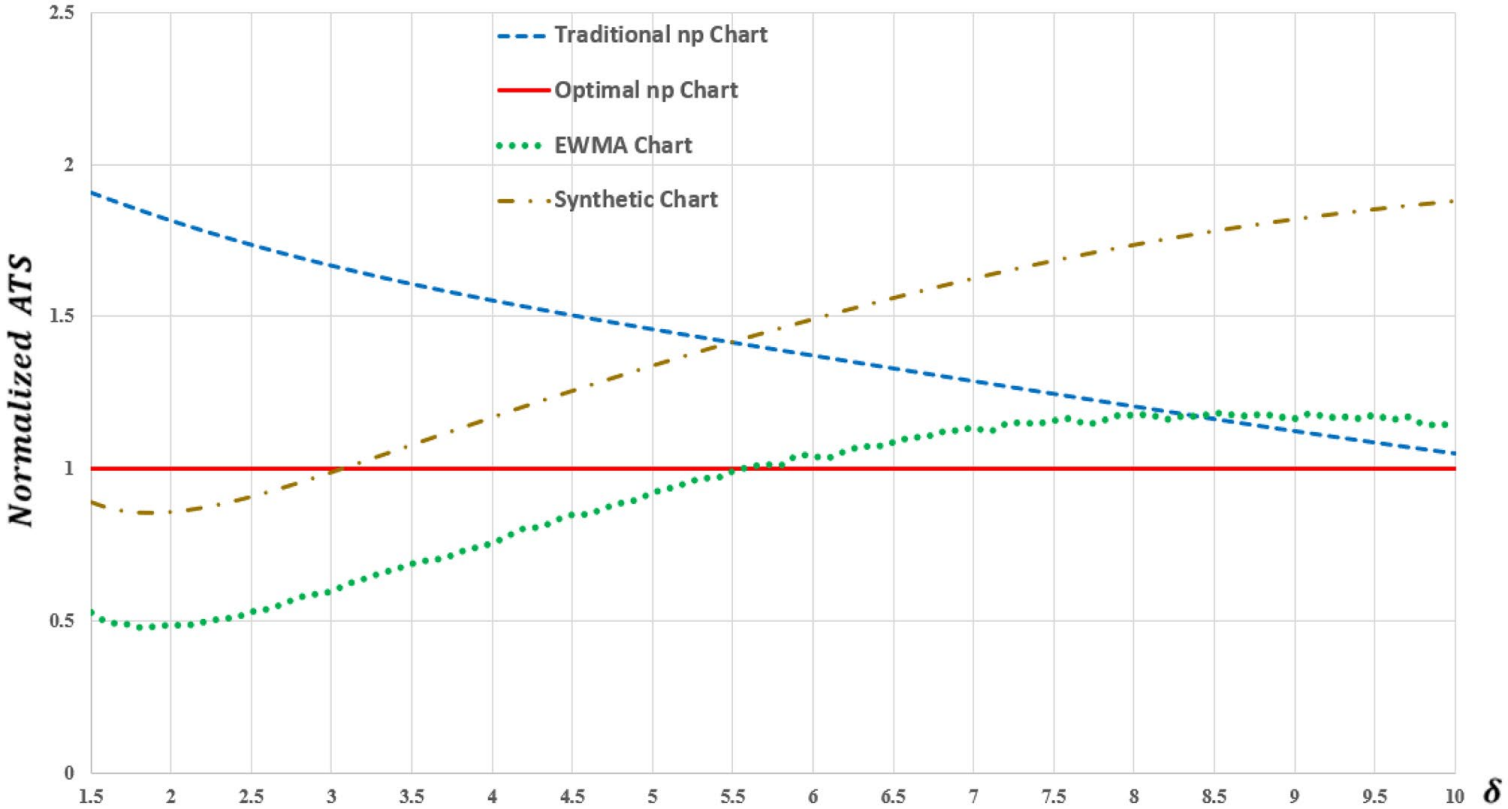
Normalized *ATS* for optimal np, traditional np, EWMA and synthetic chart.

A normalized *ATS* curves ($ATS/{ATS}_{Optimal}$) are established to compare the detection speed of the four charts over the given range of shifts, as presented in Fig. 5. Economically, the *ETC* of the optimal np chart is less than its traditional np opponent, which indicates that the optimal np chart is more economical than traditional np chart. The ratio ${ETC}_{Traditional}$/${ETC}_{Optimal}$ = 103.88/89.06 $=$ 1.17 reveals that the optimal np chart outpaces the traditional np chart by 17%, from an overall perspective. On the statistical side, the values of ${ATS}_{0}$ and out of control *ATS* are presented in Table 2.

**Table 2:** *ATS* values of optimal np, traditional np, EWMA and synthetic charts.

$\delta$	*ATS*			
	Traditional np chart	Optimal np chart	EWMA chart	Synthetic chart
1	626.498	311.810	301.719	311.54798
2	55.812	30.765	15.151	26.44278
3	15.434	9.264	5.566	9.17749
4	6.687	4.309	3.305	5.04107
5	3.674	2.521	2.309	3.37530
6	2.335	1.704	1.770	2.54143
7	1.640	1.275	1.454	2.07189
8	1.240	1.030	1.209	1.78785
9	0.993	0.884	1.039	1.60821
10	0.834	0.794	0.917	1.49157

From Fig. 5 and Table 2, the following highlights can be obtained:

1. When the process is in-control (i.e., *δ* = 1), all charts result in an ${ATS}_{0}$ that is greater than *τ*, which satisfies constraint (2) in the optimization model. This is an indication of meeting the false alarm requirement. It is also worth mentioning that constraint (3) on the inspection rate is fulfilled as *n*/*h* for both charts is equal to *r* (= 50 units per day).
2. The *ATS* values of traditional and optimal np charts indicate that the latter outperforms the former over shift range of (1 < *δ* ≤ 10). Through all shift points, the out-of-control *ATS* of the optimal np chart is always less than that of the traditional np chart. This emphasizes the superiority of the optimal np chart over the traditional np chart for detecting both small and large shifts and its ability to detect an out-of-control scenarios faster.
3. In addition, the value of *ATS* for optimal np chart is closer to the specified *τ* = 300. On the other hand, *ATS* of the traditional np chart is much larger than *τ* which indicates the lower effectiveness of the traditional np chart in identifying out-of-control conditions.
4. The optimal np chart’s dominance over the traditional np chart diminishes with increasing the shift. For example, when $\delta$ = 2, the optimal np chart detects an out-of-control signal faster than the traditional one by 81%, but when $\delta$ = 8, the former is quicker than the latter by only 20%.
5. The results suggest that the synthetic chart performs better than the optimal np chart in detecting small shifts (when *δ* < 3), whereas the EWMA chart outperforms the optimal np chart in detecting small to moderate shifts (when *δ* < 5.5). These findings align with the existing literature that indicates the superior performance of the synthetic and EWMA charts in detecting small to moderate shifts^7,9,42^. This is justifiable as the EWMA chart takes into consideration not only the latest sample but also the real-time accumulation of all samples^7,49,50^. Meanwhile, the synthetic chart, which combines the np chart with the conforming run length (CRL) chart, has been recognized as a more effective scheme for detecting small and moderate shifts compared to the np chart alone^51–53^. Notably, the synthetic chart exhibits almost identical overall performance to the optimal np chart in terms of the *ETC*. However, it is worth mentioning that the EWMA chart outperforms both the synthetic and np charts in terms of *ETC*, demonstrating a smaller cost value.

**Figure 6:**
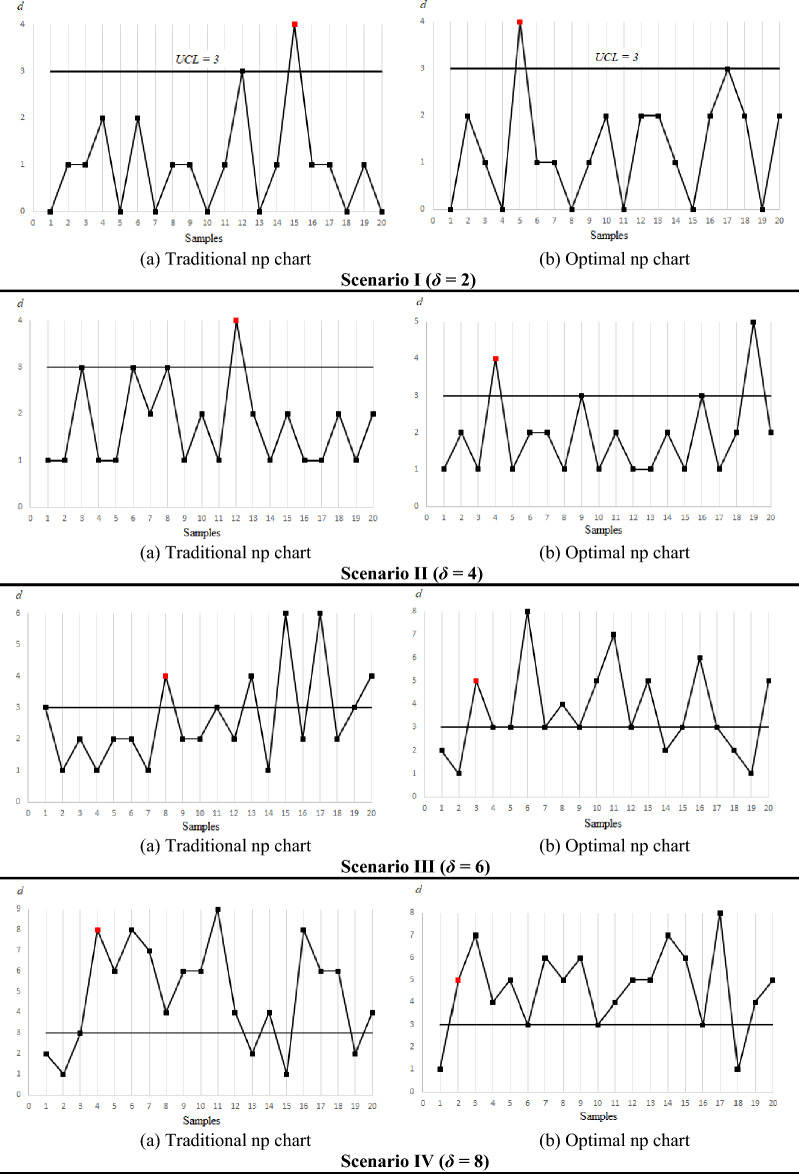
Comparison of the detection speed of the two np charts under four different scenarios.

As the developed optimal np chart represents an optimized version of the traditional np chart, an additional investigation using simulation is conducted to compare the detection speed of both charts. This assessment was carried out under various scenarios to examine their respective performance in detecting anomalies. Simulated data for the number of defective bottles (*d*_*i*_) that follows binomial distribution in 20 samples were generated using Minitab to illustrate the detection speed of the traditional and optimal np charts under four scenarios with different shift sizes (*δ* = 2, 4, 6 and 8) within the shift range of 1 < *δ* ≤ 10. As shown in Fig. 6, the optimal np chart consistently provided an out-of-control signal (plotted in red) before the traditional chart in all scenarios. This demonstrates that the former has a better detection speed than the latter. Furthermore, it can be observed that the superiority of the traditional np chart approaches that of the optimal np chart as the shift *δ* increases. This finding is consistent with the results of the *ATS* values presented in Fig. 5 and Table 2.

### Sensitivity analysis using uniform distribution

In this section, a ${2}^{12-6}$ fractional factorial design is conducted where the design specifications (${p}_{0}$, *r*, *τ*, ${\delta }_{max}$, $\lambda$, *e* and *D*) and cost parameters (*b*, *c*, *W*, *T* and *M*) are used as input factors to investigate their effect on the response, that is the expected total cost (*ETC*). Each one of the 12 factors varies at 2-levels as shown in Table 3. The levels are selected according to Refs.^22,54^. In this section, a uniform distribution is assumed for the *p* shift where the probability density function ${f}_{\updelta }\left(\updelta \right)$ is depicted in as follows:

**Table 3:** Factors levels.

Factors	Low level	High level
${p}_{0}$	0.01	0.05
${\delta }_{max}$	5	10
*τ*	300	600
$r$	50	100
*b*	0.5	1
*c*	0.01	0.09
*M*	1000	10,000
*W*	50	300
*T*	100	500
$\varepsilon$	0.01	0.05
*e*	0.025	0.125
*D*	1	3

9 $${f}_{\updelta }\left(\updelta \right)=\frac{1}{{\updelta }_{\mathit{max}}- 1}.$$

The results of the factorial design are shown in Table 4. As shown in Table 4, the performance of the traditional and the optimal np charts are compared in terms of *ETC*. The relative improvement is then calculated by ${ETC}_{Traditional}$/${ETC}_{Optimal}$ which is shown in the right-most column in Table 4 to illustrate the superiority of the optimal np chart against its opponent. It is obvious that the relative improvement is always larger than 1, which denotes that the optimal np chart always outperforms the traditional np chart in terms of *ETC*. Eventually, the grand average over the 64 runs represents the overall performance as calculated by $\overline{{ETC }_{Traditional}/{ETC}_{Optimal} }=1.45$. This percentage shows that from an overall viewpoint (considering different design specifications and cost parameters combinations), the optimal np chart effectiveness (in terms of *ETC*) exceeds its opponent, the traditional np chart by about 45%.

**Table 4:** ${2}^{12-6}$ experiment using uniform distribution as the shift distribution.

Run	Values of the input factors												Chart	Results				${ETC}_{Traditional}$/${ETC}_{Optimal}$
	${p}_{0}$	${\delta }_{max}$	*τ*	*r*	*b*	*c*	*M*	*W*	*T*	$\varepsilon$	*e*	*D*		*n*	*h*	*UCL*	*ETC*	
1	0.05	10	600	100	1	0.01	10,000	300	500	0.05	0.125	3	Traditional np	100	1	12	1320.665	2.032
													Optimal np	6	0.06	3	649.785	
2	0.05	5	600	50	1	0.01	1000	300	100	0.05	0.025	1	Traditional np	50	1	8	33.577	1.103
													Optimal np	56	1.12	8	30.446	
3	0.05	5	600	50	1	0.09	10,000	50	100	0.01	0.025	1	Traditional np	50	1	8	82.959	1.204
													Optimal np	69	1.38	9	68.927	
4	0.05	5	300	100	0.5	0.09	10,000	50	100	0.05	0.125	1	Traditional np	100	1	12	725.784	1.987
													Optimal np	14	0.14	4	365.263	
5	0.01	10	300	100	0.5	0.01	1000	50	500	0.01	0.025	3	Traditional np	100	1	5	4.693	1.263
													Optimal np	50	0.5	3	3.714	
6	0.01	10	600	100	0.5	0.01	1000	50	100	0.05	0.125	1	Traditional np	100	1	5	26.365	1.625
													Optimal np	11	0.11	2	16.220	
7	0.01	5	600	50	1	0.09	10,000	300	100	0.05	0.025	1	Traditional np	50	1	3	128.664	1.122
													Optimal np	99	1.98	4	114.627	
8	0.05	10	600	100	1	0.01	1000	300	500	0.05	0.125	3	Traditional np	100	1	12	134.989	1.897
													Optimal np	12	0.12	4	71.171	
9	0.05	10	300	100	1	0.09	10,000	300	100	0.05	0.025	3	Traditional np	100	1	12	667.764	1.314
													Optimal np	23	0.23	5	508.199	
10	0.05	10	600	100	1	0.01	10,000	50	500	0.05	0.125	3	Traditional np	100	1	12	1318.561	2.038
													Optimal np	6	0.06	3	646.836	
11	0.01	10	300	50	0.5	0.09	1000	50	100	0.01	0.025	3	Traditional np	50	1	3	4.650	1.128
													Optimal np	65	1.3	3	4.120	
12	0.01	5	300	100	0.5	0.09	1000	50	100	0.01	0.025	1	Traditional np	100	1	5	4.609	1.617
													Optimal np	99	0.99	4	2.851	
13	0.01	10	600	100	1	0.01	10,000	300	500	0.05	0.125	3	Traditional np	100	1	5	280.854	1.473
													Optimal np	11	0.11	2	190.666	
14	0.01	10	600	100	1	0.01	10,000	300	500	0.01	0.025	1	Traditional np	100	1	5	35.407	1.238
													Optimal np	80	0.8	4	28.603	
15	0.05	5	600	50	1	0.01	1000	300	100	0.05	0.125	1	Traditional np	50	1	8	58.145	1.239
													Optimal np	23	0.46	5	46.930	
16	0.01	10	300	50	1	0.01	1000	50	500	0.01	0.025	1	Traditional np	50	1	3	3.418	1.178
													Optimal np	65	1.3	3	2.902	
17	0.01	5	300	100	0.5	0.09	1000	300	500	0.01	0.125	1	Traditional np	100	1	5	7.205	1.533
													Optimal np	50	0.5	3	4.700	
18	0.05	10	300	50	0.5	0.09	1000	300	500	0.01	0.025	1	Traditional np	50	1	7	10.396	1.077
													Optimal np	26	0.52	5	9.651	
19	0.01	5	600	100	1	0.01	10,000	50	100	0.05	0.125	1	Traditional np	100	1	5	181.458	1.237
													Optimal np	39	0.39	3	146.733	
20	0.05	5	300	50	1	0.09	10,000	300	100	0.05	0.125	1	Traditional np	50	1	7	521.330	1.274
													Optimal np	16	0.32	4	409.331	
21	0.05	5	600	100	1	0.01	10,000	300	50	0.05	0.125	3	Traditional np	100	1	12	782.635	1.542
													Optimal np	20	0.2	5	507.667	
22	0.01	5	300	50	0.5	0.09	1000	50	100	0.01	0.025	1	Traditional np	50	1	3	4.543	1.496
													Optimal np	123	2.46	4	3.036	
23	0.01	5	600	50	0.5	0.09	1000	50	100	0.01	0.025	1	Traditional np	50	1	3	4.543	1.209
													Optimal np	99	1.98	4	3.759	
24	0.01	5	600	100	1	0.09	1000	300	100	0.01	0.125	1	Traditional np	100	1	5	7.216	1.313
													Optimal np	39	0.39	3	5.495	
25	0.01	5	300	50	0.5	0.01	1000	50	100	0.01	0.025	1	Traditional np	50	1	3	4.403	1.520
													Optimal np	123	2.46	4	2.896	
26	0.05	5	300	50	1	0.09	10,000	50	100	0.01	0.125	1	Traditional np	50	1	7	138.801	1.252
													Optimal np	26	0.52	5	110.824	
27	0.01	5	300	50	1	0.09	1000	50	100	0.01	0.025	1	Traditional np	50	1	3	4.561	1.499
													Optimal np	123	2.46	4	3.043	
28	0.01	10	600	50	1	0.01	10,000	50	500	0.05	0.125	3	Traditional np	50	1	3	224.852	1.129
													Optimal np	16	0.32	2	199.223	
29	0.01	10	600	100	0.5	0.01	10,000	50	100	0.05	0.025	3	Traditional np	100	1	5	160.903	1.119
													Optimal np	39	0.39	3	143.816	
30	0.01	10	300	50	0.5	0.09	1000	50	100	0.01	0.025	1	Traditional np	50	1	3	3.592	1.179
													Optimal np	65	1.3	3	3.046	
31	0.01	5	600	50	0.5	0.09	10,000	50	100	0.05	0.025	1	Traditional np	50	1	3	128.354	1.123
													Optimal np	99	1.98	4	114.316	
32	0.01	5	600	100	1	0.01	10,000	300	100	0.05	0.025	1	Traditional np	100	1	5	122.901	1.197
													Optimal np	131	1.31	5	102.711	
33	0.05	10	300	100	1	0.01	10,000	300	500	0.05	0.125	3	Traditional np	100	1	12	1320.665	2.168
													Optimal np	7	0.07	3	609.037	
34	0.05	10	300	50	0.5	0.01	10,000	300	100	0.05	0.125	3	Traditional np	50	1	7	972.900	1.521
													Optimal np	8	0.16	3	639.537	
35	0.05	5	300	50	1	0.01	10,000	50	100	0.01	0.125	3	Traditional np	50	1	7	167.283	1.192
													Optimal np	26	0.52	5	140.347	
36	0.05	5	300	50	0.5	0.01	1000	50	500	0.01	0.125	1	Traditional np	50	1	7	14.283	1.238
													Optimal np	26	0.52	5	11.533	
37	0.05	10	600	50	1	0.01	10,000	300	500	0.05	0.125	3	Traditional np	50	1	8	987.426	1.469
													Optimal np	7	0.14	3	672.269	
38	0.01	5	300	50	0.5	0.09	1000	50	100	0.01	0.025	1	Traditional np	50	1	3	4.527	1.494
													Optimal np	123	2.46	4	3.030	
39	0.05	5	600	100	1	0.09	10,000	300	100	0.01	0.025	1	Traditional np	100	1	12	71.832	1.125
													Optimal np	62	0.62	9	63.829	
40	0.01	5	300	50	1	0.09	10,000	300	500	0.01	0.025	1	Traditional np	50	1	3	43.788	1.522
													Optimal np	123	2.46	4	28.770	
41	0.05	10	600	100	1	0.01	10,000	300	500	0.05	0.025	3	Traditional np	100	1	12	665.501	1.277
													Optimal np	20	0.2	5	520.951	
42	0.01	5	300	50	1	0.01	1000	300	500	0.05	0.025	3	Traditional np	50	1	3	14.611	1.198
													Optimal np	123	2.46	4	12.194	
43	0.05	10	600	100	0.5	0.01	10,000	300	500	0.05	0.125	3	Traditional np	100	1	12	1320.515	2.040
													Optimal np	6	0.06	3	647.285	
44	0.01	5	300	50	0.5	0.09	10,000	50	100	0.01	0.025	1	Traditional np	50	1	3	43.674	1.525
													Optimal np	123	2.46	4	28.640	
45	0.01	10	600	50	1	0.09	10,000	300	100	0.01	0.025	3	Traditional np	50	1	3	43.636	1.015
													Optimal np	99	1.98	4	42.981	
46	0.05	10	600	50	0.5	0.01	1000	300	100	0.05	0.025	1	Traditional np	50	1	8	40.656	1.149
													Optimal np	23	0.46	5	35.376	
47	0.05	10	600	100	1	0.01	10,000	300	500	0.01	0.125	3	Traditional np	100	1	12	407.895	2.408
													Optimal np	12	0.12	4	169.425	
48	0.05	10	300	50	0.5	0.09	1000	50	100	0.05	0.125	3	Traditional np	50	1	7	99.035	1.489
													Optimal np	8	0.16	3	66.503	
49	0.05	10	600	100	1	0.01	10,000	300	100	0.05	0.125	3	Traditional np	100	1	12	1320.566	2.033
													Optimal np	6	0.06	3	649.650	
50	0.05	10	600	100	1	0.09	10,000	300	500	0.05	0.125	3	Traditional np	100	1	12	1323.065	2.029
													Optimal np	6	0.06	3	652.185	
51	0.05	5	300	100	1	0.09	10,000	50	100	0.01	0.125	1	Traditional np	100	1	12	218.651	2.243
													Optimal np	14	0.14	4	97.479	
52	0.05	10	600	100	1	0.01	1000	300	100	0.01	0.025	3	Traditional np	100	1	12	17.579	1.196
													Optimal np	29	0.29	6	14.694	
53	0.05	10	600	100	1	0.09	1000	300	500	0.01	0.125	1	Traditional np	100	1	12	40.054	2.305
													Optimal np	12	0.12	4	17.376	
54	0.01	5	300	50	1	0.09	10,000	300	100	0.05	0.125	1	Traditional np	50	1	3	160.846	1.135
													Optimal np	23	0.46	2	141.763	
55	0.01	10	300	50	1	0.01	1000	50	100	0.05	0.125	3	Traditional np	50	1	3	22.654	1.206
													Optimal np	23	0.46	2	18.784	
56	0.01	10	300	100	1	0.01	1000	50	500	0.01	0.125	3	Traditional np	100	1	5	9.431	1.790
													Optimal np	16	0.16	2	5.268	
57	0.05	10	300	100	1	0.01	1000	300	100	0.05	0.025	3	Traditional np	100	1	12	70.265	1.267
													Optimal np	23	0.23	5	55.472	
58	0.01	10	600	100	1	0.09	1000	50	100	0.01	0.025	3	Traditional np	100	1	5	5.191	1.141
													Optimal np	80	0.8	4	4.549	
59	0.05	10	600	50	1	0.09	10,000	300	100	0.01	0.125	1	Traditional np	50	1	8	223.001	1.777
													Optimal np	14	0.28	4	125.470	
60	0.05	5	600	100	0.5	0.09	10,000	300	500	0.01	0.025	3	Traditional np	100	1	12	103.501	1.081
													Optimal np	62	0.62	9	95.762	
61	0.01	5	600	100	1	0.01	10,000	300	100	0.01	0.125	3	Traditional np	100	1	5	72.504	1.311
													Optimal np	39	0.39	3	55.287	
62	0.05	5	600	100	0.5	0.09	1000	50	500	0.05	0.025	3	Traditional np	100	1	12	42.964	1.080
													Optimal np	50	0.5	8	39.768	
63	0.01	5	300	50	0.5	0.01	1000	50	100	0.05	0.125	1	Traditional np	50	1	3	16.122	1.133
													Optimal np	23	0.46	2	14.236	
64	0.01	5	600	100	0.5	0.09	1000	50	500	0.05	0.125	3	Traditional np	100	1	5	19.332	1.189
													Optimal np	39	0.39	3	16.263	

**Figure 7:**
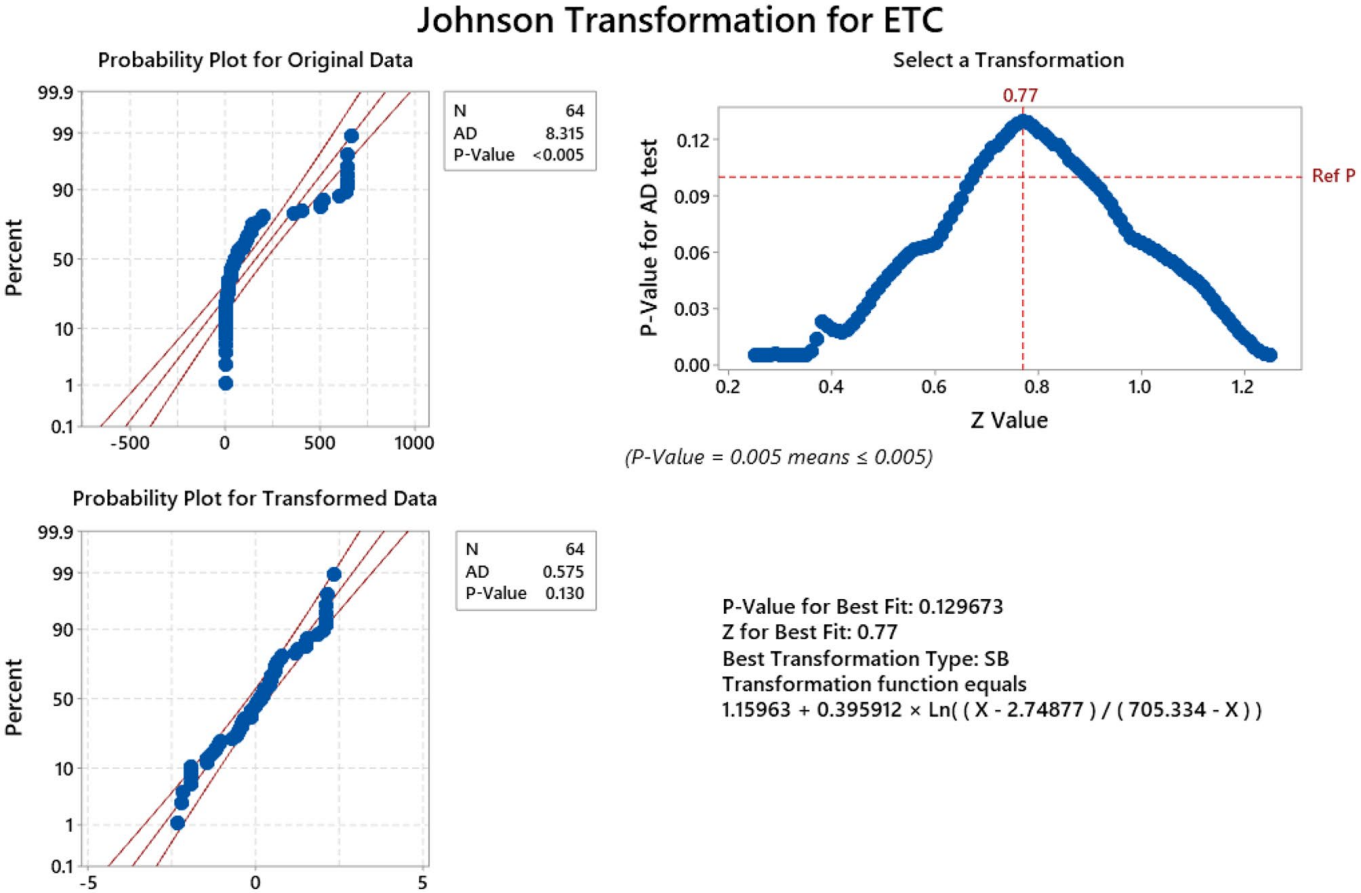
Normality test and transformation of *ETC* for optimal np chart data.

To identify the factors that affect the performance of the optimal np chart significantly, an Analysis of Variance (ANOVA) is conducted at 5% level of significance. However, the data on the *ETC* of the optimal np chart should be normally distributed in order to perform ANOVA. Thus, a normality test is performed on the data of the *ETC* values of the optimal np chart. However, the original *ETC* values are not normally distributed as shown in Fig. 7 as the p-value is smaller than 0.05, hence Johnson transformation is performed to ensure the normality of *ETC* values, and it is found that p-value of 0.129 is produced after the transformation which emphasizes that the transformed *ETC* values are normally distributed. ANOVA results for the main effects are summarized in Table 5. It shows the effects of the input factors on *ETC* of the optimal np chart and the corresponding p-value. Since the replication size is 1, the high order interactions (third order and higher) are combined to determine the sum of square errors. Table 5 shows that there are three significant input factors with p-value smaller than 0.05, namely ${p}_{0}$, $\varepsilon$ and *M.* The *ETC* of an optimal np chart is positively influenced by $\varepsilon$(p-value $=0.001$), implying that a higher $\varepsilon$ would result in a higher *ETC* and vice versa. On the other hand, ${p}_{0}$ (p-value $=0.004$) and *M* (p-value $=0.0001$) have a negative impact on the *ETC*, implying that a bigger ${p}_{0}$ or *M* lowers the *ETC* and vice versa.

**Table 5:** Effect of input factors using ANOVA. Significant factors are in bold.

Input factors	Effect on *ETC* of optimal np chart	p-value
${{\varvec{p}}}_{0}$	** − 54.8**	**0.004**
${\delta }_{max}$	** − **37.2	0.065
*τ*	29.3	0.127
*r*	** − **10.5	0.584
*b*	18.3	0.369
*c*	** − **2.9	0.881
***M***	** − 106.4**	**0.0001**
*W*	** − **9.0	0.661
*T*	** − **18.8	0.328
${\varvec{\varepsilon}}$	**70**	**0.001**
*D*	** − **21.5	0.250
*e*	** − **19.8	0.365

### Sensitivity analysis using Rayleigh distribution

In many SPC research, uniform distribution was assumed to represent the *p* shift^22,23,30^. However, in reality, the *p* shift is not predictable and can follow any probability distribution. In this study, the random shift $\delta$ is assumed to follow a uniform distribution where the probability of the occurrence of any shift size over the range of interest is equal. However, it is worthwhile to use another probability distribution such as Rayleigh distribution to describe the *p* shift in order to reach a general conclusion about the performance of the proposed optimal np chart under different shift distributions. Rayleigh distribution gives less weight to large shifts, and consequently, it is more realistic to represent the process shift in practice. Rayleigh distribution is used to characterize the potential deviation from the target in geometric tolerance. The probability density function ${f}_{\delta }(\delta )$ and the cumulative distribution function $F(\delta )$ of the Rayleigh distribution are denoted as follows:

10 $${f}_{\delta }\left(\delta \right)=\frac{\pi \left(\delta -1\right)}{{2\left({\mu }_{\delta }-1\right)}^{2}}{\text{exp}}\left(\frac{\pi {\left(\delta -1\right)}^{2}}{{4\left({\mu }_{\delta }-1\right)}^{2}}\right),$$

11 $$F\left(\delta \right)=1-{\text{exp}}\left(\frac{-\pi {\left(\delta -1\right)}^{2}}{{4\left({\mu }_{\delta }-1\right)}^{2}}\right),$$

where $\delta$ is the increasing *p* shift (1 < *δ* ≤ ${\delta }_{max}$), ${\mu }_{\delta }$ is the mean of *δ* and ${\delta }_{max}$ is the maximum value of $\delta$. To facilitate the comparison of both distributions, ${\mu }_{\delta }$ of the Rayleigh distribution is calculated based on ${\delta }_{max}$ and ${p}_{0}$ of the uniform distribution such that the probability of* p* $>{p}_{max}$ is negligible (lower than 0.001) as follows:

12 $${\mu }_{\delta }={p}_{0}+\sqrt{\frac{{- \pi {p}_{0} ({\delta }_{max}- 1)}^{2}}{4\,{\text{ln}}\,0.001}}.$$

The 64 cases in Table 6 generated by the ${2}^{12-6}$ fractional factorial design shows the comparison of the traditional and optimal np charts while the shift follows a Rayleigh distribution. It is clear from Table 6 that the *ETC* of the traditional np chart exceeds the *ETC* of the optimal np chart for all 64 runs. This indicates the superiority of the optimal np chart against its opponent, as reflected by the relative improvement calculated in the right-most column of Table 6. Lastly, the grand average that represents the overall performance over 64 runs is calculated as $\overline{{ETC }_{Traditional}/{ETC}_{Optimal} }=1.30$, which indicates the overall superiority of the optimal np chart against its opponent through all 64 runs. The results of “Sensitivity analysis using uniform distribution” and “Sensitivity analysis using Rayleigh distribution” sections pinpoint that the optimal np chart always outperforms its counterpart, regardless of the distribution of the *p* shift.

**Table 6:** ${2}^{12-6}$ experiment using Rayleigh distribution as the shift distribution.

Run	Values of the input factors												Chart	Results				${ETC}_{Traditional}$/${ETC}_{Optimal}$
	${p}_{0}$	${\mu }_{\delta }$	*τ*	*r*	*b*	*c*	*M*	*W*	*T*	$\varepsilon$	*e*	*D*		*n*	*h*	*UCL*	*ETC*	
1	0.05	0.217	600	100	1	0.01	10,000	300	500	0.05	0.125	3	Traditional np	100	1	12	897.269	1.683
													Optimal np	12	0.12	4	533.107	
2	0.05	0.117	600	50	1	0.01	1000	300	100	0.05	0.025	1	Traditional np	50	1	8	42.420	1.273
													Optimal np	110	2.2	12	33.329	
3	0.05	0.117	600	50	1	0.09	10,000	50	100	0.01	0.025	1	Traditional np	50	1	8	158.374	1.609
													Optimal np	125	2.5	13	98.439	
4	0.05	0.117	300	100	0.5	0.09	10,000	50	100	0.05	0.125	1	Traditional np	100	1	12	542.795	1.372
													Optimal np	23	0.23	5	395.680	
5	0.01	0.040	300	100	0.5	0.01	1000	50	500	0.01	0.025	3	Traditional np	100	1	5	4.621	1.390
													Optimal np	99	0.99	4	3.324	
6	0.01	0.040	600	100	0.5	0.01	1000	50	100	0.05	0.125	1	Traditional np	100	1	5	19.659	1.298
													Optimal np	39	0.39	3	15.147	
7	0.01	0.023	600	50	1	0.09	10,000	300	100	0.05	0.025	1	Traditional np	50	1	3	140.226	1.118
													Optimal np	99	1.98	4	125.435	
8	0.05	0.202	600	100	1	0.01	1000	300	500	0.05	0.125	3	Traditional np	100	1	12	91.671	1.605
													Optimal np	12	0.12	4	57.105	
9	0.05	0.202	300	100	1	0.09	10,000	300	100	0.05	0.025	3	Traditional np	100	1	12	467.710	1.201
													Optimal np	33	0.33	6	389.433	
10	0.05	0.202	600	100	1	0.01	10,000	50	500	0.05	0.125	3	Traditional np	100	1	12	895.873	1.686
													Optimal np	12	0.12	4	531.253	
11	0.01	0.040	300	50	0.5	0.09	1000	50	100	0.01	0.025	3	Traditional np	50	1	3	4.822	1.290
													Optimal np	123	2.46	4	3.737	
12	0.01	0.024	300	100	0.5	0.09	1000	50	100	0.01	0.025	1	Traditional np	100	1	5	6.967	1.863
													Optimal np	99	0.99	4	3.741	
13	0.01	0.040	600	100	1	0.01	10,000	300	500	0.05	0.125	3	Traditional np	100	1	5	206.367	1.243
													Optimal np	39	0.39	3	165.966	
14	0.01	0.040	600	100	1	0.01	10,000	300	500	0.01	0.025	1	Traditional np	100	1	5	38.977	1.312
													Optimal np	131	1.31	5	29.706	
15	0.05	0.117	600	50	1	0.01	1000	300	100	0.05	0.125	1	Traditional np	50	1	8	53.867	1.097
													Optimal np	33	0.66	6	49.106	
16	0.01	0.040	300	50	1	0.01	1000	50	500	0.01	0.025	1	Traditional np	50	1	3	4.053	1.375
													Optimal np	123	2.46	4	2.949	
17	0.01	0.023	300	100	0.5	0.09	1000	300	500	0.01	0.125	1	Traditional np	100	1	5	8.202	1.561
													Optimal np	50	0.5	3	5.254	
18	0.05	0.202	300	50	0.5	0.09	1000	300	500	0.01	0.025	1	Traditional np	50	1	7	8.362	1.008
													Optimal np	38	0.76	6	8.294	
19	0.01	0.023	600	100	1	0.01	10,000	50	100	0.05	0.125	1	Traditional np	100	1	5	158.184	1.130
													Optimal np	80	0.8	4	139.960	
20	0.05	0.117	300	50	1	0.09	10,000	300	100	0.05	0.125	1	Traditional np	50	1	7	447.019	1.039
													Optimal np	38	0.76	6	430.034	
21	0.05	0.117	600	100	1	0.01	10,000	300	50	0.05	0.125	3	Traditional np	100	1	12	575.798	1.167
													Optimal np	29	0.29	6	493.330	
22	0.01	0.023	300	50	0.5	0.09	1000	50	100	0.01	0.025	1	Traditional np	50	1	3	5.958	1.382
													Optimal np	108	2.16	4	4.311	
23	0.01	0.023	600	50	0.5	0.09	1000	50	100	0.01	0.025	1	Traditional np	50	1	3	6.499	1.208
													Optimal np	99	1.98	4	5.382	
24	0.01	0.023	600	100	1	0.09	1000	300	100	0.01	0.125	1	Traditional np	100	1	5	8.211	1.297
													Optimal np	80	0.8	4	6.332	
25	0.01	0.023	300	50	0.5	0.01	1000	50	100	0.01	0.025	1	Traditional np	50	1	3	6.405	1.644
													Optimal np	123	2.46	4	3.896	
26	0.05	0.117	300	50	1	0.09	10,000	50	100	0.01	0.125	1	Traditional np	50	1	7	142.729	1.032
													Optimal np	38	0.76	6	138.243	
27	0.01	0.023	300	50	1	0.09	1000	50	100	0.01	0.025	1	Traditional np	50	1	3	6.511	1.630
													Optimal np	123	2.46	4	3.995	
28	0.01	0.202	600	50	1	0.01	10,000	50	500	0.05	0.125	3	Traditional np	50	1	8	704.151	1.255
													Optimal np	14	0.28	4	561.154	
29	0.01	0.040	600	100	0.5	0.01	10,000	50	100	0.05	0.025	3	Traditional np	100	1	5	138.669	1.121
													Optimal np	80	0.8	4	123.662	
30	0.01	0.040	300	50	0.5	0.09	1000	50	100	0.01	0.025	1	Traditional np	50	1	3	4.171	1.367
													Optimal np	123	2.46	4	3.052	
31	0.01	0.023	600	50	0.5	0.09	10,000	50	100	0.05	0.025	1	Traditional np	50	1	3	140.097	1.118
													Optimal np	99	1.98	4	125.292	
32	0.01	0.023	600	100	1	0.01	10,000	300	100	0.05	0.025	1	Traditional np	100	1	5	139.267	1.262
													Optimal np	131	1.31	5	110.331	
33	0.05	0.202	300	100	1	0.01	10,000	300	500	0.05	0.125	3	Traditional np	100	1	12	897.269	1.805
													Optimal np	14	0.14	4	497.233	
34	0.05	0.202	300	50	0.5	0.01	10,000	300	100	0.05	0.125	3	Traditional np	50	1	7	677.842	1.275
													Optimal np	16	0.32	4	531.480	
35	0.05	0.117	300	50	1	0.01	10,000	50	100	0.01	0.125	3	Traditional np	50	1	7	160.205	1.028
													Optimal np	38	0.76	6	155.898	
36	0.05	0.117	300	50	0.5	0.01	1000	50	500	0.01	0.125	1	Traditional np	50	1	7	14.533	1.030
													Optimal np	38	0.76	6	14.105	
37	0.05	0.202	600	50	1	0.01	10,000	300	500	0.05	0.125	3	Traditional np	50	1	8	705.787	1.254
													Optimal np	0.14	0.28	4	562.970	
38	0.01	0.023	300	50	0.5	0.09	1000	50	100	0.01	0.025	1	Traditional np	50	1	3	6.499	1.629
													Optimal np	123	2.46	4	3.990	
39	0.05	0.117	600	100	1	0.09	10,000	300	100	0.01	0.025	1	Traditional np	100	1	12	93.055	1.034
													Optimal np	115	1.15	13	89.968	
40	0.01	0.117	300	50	1	0.09	10,000	300	500	0.01	0.025	1	Traditional np	50	1	7	95.339	1.137
													Optimal np	106	2.12	11	83.831	
41	0.05	0.202	600	100	1	0.01	10,000	300	500	0.05	0.025	3	Traditional np	100	1	12	466.191	1.142
													Optimal np	39	0.39	7	408.124	
42	0.01	0.023	300	50	1	0.01	1000	300	500	0.05	0.025	3	Traditional np	50	1	3	14.605	1.263
													Optimal np	123	2.46	4	11.561	
43	0.05	0.202	600	100	0.5	0.01	10,000	300	500	0.05	0.125	3	Traditional np	100	1	12	897.169	1.686
													Optimal np	12	0.12	4	532.268	
44	0.01	0.023	300	50	0.5	0.09	10,000	50	100	0.01	0.025	1	Traditional np	50	1	3	63.832	1.647
													Optimal np	123	2.46	4	38.761	
45	0.01	0.040	600	50	1	0.09	10,000	300	100	0.01	0.025	3	Traditional np	50	1	3	46.302	1.137
													Optimal np	99	1.98	4	40.725	
46	0.05	0.202	600	50	0.5	0.01	1000	300	100	0.05	0.025	1	Traditional np	50	1	8	33.642	1.077
													Optimal np	44	0.88	7	31.239	
47	0.05	0.202	600	100	1	0.01	10,000	300	500	0.01	0.125	3	Traditional np	100	1	12	281.441	1.884
													Optimal np	20	0.2	5	149.379	
48	0.05	0.202	300	50	0.5	0.09	1000	50	100	0.05	0.125	3	Traditional np	50	1	7	68.942	1.265
													Optimal np	16	0.32	4	54.510	
49	0.05	0.202	600	100	1	0.01	10,000	300	100	0.05	0.125	3	Traditional np	100	1	12	897.204	1.683
													Optimal np	12	0.12	4	533.016	
50	0.05	0.202	600	100	1	0.09	10,000	300	500	0.05	0.125	3	Traditional np	100	1	12	898.879	1.681
													Optimal np	12	0.12	4	534.716	
51	0.05	0.117	300	100	1	0.09	10,000	50	100	0.01	0.125	1	Traditional np	100	1	12	184.819	1.457
													Optimal np	33	0.33	6	126.838	
52	0.05	0.202	600	100	1	0.01	1000	300	100	0.01	0.025	3	Traditional np	100	1	12	12.195	1.097
													Optimal np	50	0.50	8	11.112	
53	0.05	0.202	600	100	1	0.09	1000	300	500	0.01	0.125	1	Traditional np	100	1	12	27.672	1.876
													Optimal np	20	0.20	5	14.754	
54	0.01	0.023	300	50	1	0.09	10,000	300	100	0.05	0.125	1	Traditional np	50	1	3	150.252	1.119
													Optimal np	65	1.3	3	134.227	
55	0.01	0.040	300	50	1	0.01	1000	50	100	0.05	0.125	3	Traditional np	50	1	3	18.107	1.130
													Optimal np	23	0.46	2	16.029	
56	0.01	0.040	300	100	1	0.01	1000	50	500	0.01	0.125	3	Traditional np	100	1	5	7.589	1.500
													Optimal np	16	0.16	2	5.059	
57	0.05	0.202	300	100	1	0.01	1000	300	100	0.05	0.025	3	Traditional np	100	1	12	49.083	1.175
													Optimal np	33	0.33	6	41.756	
58	0.01	0.040	600	100	1	0.09	1000	50	100	0.01	0.025	3	Traditional np	100	1	5	4.955	1.224
													Optimal np	133	1.31	5	4.049	
59	0.05	0.202	600	50	1	0.09	10,000	300	100	0.01	0.125	1	Traditional np	50	1	8	174.064	1.375
													Optimal np	23	0.46	5	126.571	
60	0.05	0.117	600	100	0.5	0.09	10,000	300	500	0.01	0.025	3	Traditional np	100	1	12	112.815	1.027
													Optimal np	115	1.15	3	109.819	
61	0.01	0.023	600	100	1	0.01	10,000	300	100	0.01	0.125	3	Traditional np	100	1	5	81.563	1.292
													Optimal np	80	0.80	4	63.112	
62	0.05	0.117	600	100	0.5	0.09	1000	50	500	0.05	0.025	3	Traditional np	100	1	12	37.429	1.010
													Optimal np	88	0.88	11	37.049	
63	0.01	0.023	300	50	0.5	0.01	1000	50	100	0.05	0.125	1	Traditional np	50	1	3	15.045	1.119
													Optimal np	65	1.3	3	13.444	
64	0.01	0.023	600	100	0.5	0.09	1000	50	500	0.05	0.125	3	Traditional np	100	1	5	16.341	1.115
													Optimal np	80	0.80	4	14.651	

## Conclusions

This research proposes an economic-statistical model for the optimal design of np control chart. The developed optimal np chart is compared with the traditional np, EWMA and synthetic control charts. In the proposed model, the decision variables, which are the sample size (*n*), sampling interval (*h*), and upper control limit (*UCL*), are optimized such that the expected total cost (*ETC*) is minimized while ensuring that the constraints on the inspection rate and the false alarm are satisfied. The effectiveness of the optimal np scheme is demonstrated by a real-life data in water bottle manufacturing. It is found that the optimal np chart economically exceeds its opponent by 17% and statistically always detect an out-of-control signal faster than the traditional np chart over the entire shift range. The proposed optimal np scheme is compared with its traditional version under different design specifications using different probability distributions of the shift. The sensitivity analysis is conducted to evaluate the impact of design specifications on the overall performance of the optimal np chart. It is found that ${p}_{0}$, $\varepsilon$ and *M* significantly affect* ETC.*

### Theoretical contribution

The main theoretical contribution of our research lies in the development of an economic-statistical optimization algorithm for the np chart. This algorithm effectively identifies the optimal charting parameters for the np chart to minimize the expected total cost (*ETC*) while adhering to statistical constraints on inspection rate and false alarm rate, ensuring that practical considerations are incorporated. Additionally, our study addresses the gap in the literature by focusing on the economic-statistical design of attribute control charts, particularly the np chart. Attribute control charts have wide-ranging applications and are easier to manage in manufacturing processes. However, they have received less attention in terms of economic-statistical design compared to variable control charts. Therefore, our research fills this gap by providing a comprehensive framework for optimizing the np chart’s statistical performance and cost efficiency. The study also conducts a sensitivity analysis to provide insights into the impact of changes in the design parameters on the performance of the np chart. By identifying the significant factors that influence the expected total cost, the study offers valued guidance for practitioners in designing effective control charts tailored to their specific process characteristics. By achieving enhanced cost efficiency and detection effectiveness, this research considerably contributes to the theoretical understanding of control chart design and optimization, thus expanding the existing body of literature in the field of statistical process control.

### Practical implications

The findings of this research hold significant practical implications for manufacturing organizations and service sectors. The developed optimal np chart presents a practical and cost-effective alternative to the traditional np chart, offering substantial benefits to quality control practitioners. By optimizing *n*,* h*, and *UCL*, practitioners can achieve significant cost savings while ensuring effective process monitoring. Moreover, this study emphasizes the importance of striking a balance between statistical properties and financial considerations in the design of control charts. By considering both aspects, industries can promptly respond to process shifts, take appropriate actions to maintain product quality, and minimize costs. The code developed in this study allows practitioners to input their desired design specifications, enabling the optimization of np chart parameters on a personal computer within seconds. This streamlines the implementation process, eliminating the need for extensive computational expertise and facilitating improved process control. The code can be obtained from authors upon request. Companies can leverage this tool to make data-driven decisions regarding process monitoring and quality control. By considering economic and statistical factors, practitioners can tailor the np chart design to suit their specific operational requirements, leading to cost reduction and overall enhanced product quality. Additionally, this research contributes to the investigating the performance of the optimal np chart under different scenarios. The insights gained provide practitioners with valuable guidelines to enhance their quality control design and achieve superior process control outcomes.

### Future research

Future research might be conducted to study the performance of the optimal np chart when ${p}_{0}$ is estimated and not known or when *d* follows Poisson distribution. In addition, the proposed economic-statistical model might be used to optimize other attribute charts such as EWMA and cumulative sum (CUSUM) charts. To further enhance the np chart’s capabilities, an important area for future exploration is the development of an optimal economic-statistical design that incorporates variable sample size and sampling interval (VSSI), curtailment, and other related features. However, integrating these aspects into the optimization process of the np control chart can introduce computational burdens for practitioners. To mitigate this challenge, it becomes crucial to consider novel technologies that can address such computational complexities. Adaptive sampling systems with switching mechanisms and efficient dual sampling systems are two promising approaches that warrant investigation^55–57^. These technologies have demonstrated the potential to alleviate computational burdens and enhance the practicality and effectiveness of the np chart optimization process. By incorporating these advancements, practitioners across various industries can benefit from improved decision-making and enhanced quality control.

## Data Availability

The datasets used and/or analyzed during the current study available from the corresponding author on reasonable request.
